# Pacsin2 is required for endocytosis in the zebrafish pronephric tubule

**DOI:** 10.1242/bio.059150

**Published:** 2022-06-23

**Authors:** Joseph Morgan, Rebecca Yarwood, Tobias Starborg, Guanhua Yan, Martin Lowe

**Affiliations:** School of Biological Sciences, Faculty of Biology, Medicine and Health, University of Manchester, The Michael Smith Building, Oxford Road, Manchester M13 9PT, UK

**Keywords:** Endocytosis, Proximal tubule, Pacsin2, Megalin, Zebrafish

## Abstract

Endocytosis mediates the cellular uptake of numerous molecules from the extracellular space and is a fundamentally important process. In the renal proximal tubule, the scavenger receptor megalin and its co-receptor cubilin mediate endocytosis of low molecular weight proteins from the renal filtrate. However, the extent to which megalin endocytosis relies on different components of the trafficking machinery remains relatively poorly defined *in vivo*. In this study, we identify a functional requirement for the F-BAR protein pacsin2 in endocytosis in the renal proximal tubule of zebrafish larvae. Pacsin2 is expressed throughout development and in all zebrafish tissues, similar to the mammalian orthologue. Within renal tubular epithelial cells, pacsin2 is enriched at the apical pole where it is localised to endocytic structures. Loss of pacsin2 results in reduced endocytosis within the proximal tubule, which is accompanied by a reduction in the abundance of megalin and endocytic organelles. Our results indicate that pacsin2 is required for efficient endocytosis in the proximal tubule, where it likely cooperates with other trafficking machinery to maintain endocytic uptake and recycling of megalin.

## Abstract (precis)

**Summary:** We identify a role for the F-BAR protein pacsin2 in endocytosis in the renal tubule of zebrafish larvae.

## INTRODUCTION

The proximal tubule is responsible for the uptake of numerous solutes and water from the renal filtrate. Low molecular weight proteins are retrieved by endocytosis from the apical pole of renal proximal tubule cells, a process mediated by the abundant scavenger receptor megalin (also known as LDL receptor related protein 2, LRP2) and its co-receptor cubulin (Christensen et al., 2012; Eshbach and Weisz, 2017). These receptors undergo cycles of internalisation and recycling to facilitate the efficient capture of numerous ligands, which dissociate in apical endosomes and are subsequently delivered to the lysosome or undergo transcytosis. Endocytic uptake is mediated by clathrin and associated adaptors and accessory proteins (Christensen et al., 2012), most notably Dab2 (Long et al., 2021; Oleinikov et al., 2000), while recycling is relatively poorly defined in molecular terms. Recycling in proximal tubular cells occurs via recycling tubules that emanate from apical early endosomes and apical vacuoles, which appear to function as a major sorting and recycling compartment in this cell type (Birn et al., 1993; Eshbach and Weisz, 2017; Hatae et al., 1997). In line with a role in recycling, apical vacuoles are associated with Rab11, a marker of recycling endosomes in other cell types (Mattila et al., 2014). Mutation of megalin is responsible for Donnai–Barrow syndrome, with characteristic tissue-specific defects including the hallmark trait of low molecular weight (LMW) proteinuria (Kantarci et al., 2007).

The PACSIN (also known as syndapin) family comprises three members in mammals, namely PACSIN1, which is neuronally expressed; PACSIN2, which is ubiquitous; and PACSIN3, which is expressed in skeletal muscle and heart (Kessels and Qualmann, 2004; Quan and Robinson, 2013). PACSINs contain an amino-terminal banana-shaped F-BAR (Fes-CIP4 homology Bin-amphiphysin-Rvs161/167) domain that binds to membranes to induce or sense membrane curvature (Frost et al., 2009), and a carboxy-terminal SH3 domain that interacts with various associated proteins, including trafficking components and actin machinery (Modregger et al., 2000; Qualmann and Kelly, 2000). PACSIN1 and PACSIN2 also contain NPF motifs that bind to EHD domain proteins (Braun et al., 2005; Xu et al., 2004), which are involved in membrane sculpting and fission and associated with caveolae formation and endocytic recycling (Naslavsky and Caplan, 2011). Pacsins have been implicated in a number of cellular processes that involve membrane remodelling, including clathrin and non-clathrin dependent endocytosis, caveolae formation, endocytic recycling, ciliogenesis, microvilli formation and neuronal morphogenesis (reviewed in Quan and Robinson, 2013). Many of these functions have been determined using *in vitro* studies, while the roles of PACSINs have been less well studied *in vivo*. In particular, animal models for the ubiquitously expressed PACSIN2 have only recently been described. Loss of PACSIN2 in mice does not affect viability or fertility and the animals appear generally healthy (Malinova et al., 2021; Postema et al., 2019; Semmler et al., 2018). However, a number of tissue-specific phenotypes have been reported in the PACSIN2 knockouts, namely effects upon microvillar structure in the intestine (Postema et al., 2019), reduced blood vessel sprouting in the retina (Malinova et al., 2021), and delayed cardiomyocyte development (Semmler et al., 2018). The former two phenotypes were attributed to endocytic trafficking defects, either defective endocytic vesicle formation at the apical membrane (Malinova et al., 2021), or defective cadherin trafficking (Malinova et al., 2021), respectively. Loss of Pacsin2 in zebrafish crispant embryos results in defective ciliogenesis in the olfactory placode, likely caused by defects in membrane tubule formation at the ciliary pocket (Insinna et al., 2019).

To better understand the *in vivo* requirements for Pacsin2, we generated a stable *pacsin2*-knockout zebrafish model using CRISPR/Cas9. We find that stable loss of Pacsin2 in zebrafish does not affect gross morphology, viability or fertility, similar to what is seen in the PACSIN2 knockout mouse. Analysis of the zebrafish pronephros, the larval kidney, revealed a defect in proximal tubular endocytosis upon loss of Pacsin2. This functional defect was accompanied by a reduction in the abundance of megalin, as well a loss of apical endocytic organelles from the proximal tubular cells. Our results indicate a role for Pacsin2 in endocytosis and maintenance of the apical endocytic apparatus in the renal proximal tubule.

## RESULTS

### Conservation of the Pacsin family in zebrafish

A previous study identified six *pacsin* orthologues in zebrafish (Edeling et al., 2009). Using sequence alignment, we too identified the six orthologues, which are shown in Fig. S1A. *pacsin1* has been duplicated in zebrafish resulting in two paralogues, named *pacsin1a* and *pacsin1b*. Synteny analysis supports the view these were generated by duplication (Fig. S1B). *pacsin2* is present as a single gene in zebrafish, whereas *pacsin3* appears have undergone a duplication event to generate an additional paralogue *ch211-51c14.1*, which in turn also appears to have been duplicated to generate a third related gene *zgc:91999* (Fig. S1A). *ch211-51c14.1* and *zgc:91999* share lower homology to mammalian *PACSIN3* than the zebrafish gene annotated as *pacsin3* (Fig. S1A). There is good conservation of the zebrafish Pacsins with their mammalian orthologues in the F-BAR and SH3 domains, with the retention of key features such as the wedge-loop in the F-BAR domain and the PxxP motif binding pocket in the SH3 domain (Fig. S1C,D). Zebrafish Pacsin1a and Pacsin1b each contain one EHD domain protein-binding NPF motif, as in mammalian PACSIN1, although in Pacsin1a the central proline residue is instead a serine, the significance of which is unclear (Fig. S1E). Pacsin2 has three NPF motifs, the same as in mammals, and the three Pacsin3 orthologues all lack NPF motifs, as is the case in the mammalian protein (Fig. S1E). Hence, there is good conservation of the zebrafish Pacsins with their mammalian counterparts, particularly in the case of Pacsin2.

### Pacsin tissue and developmental expression

**Fig. 1.**
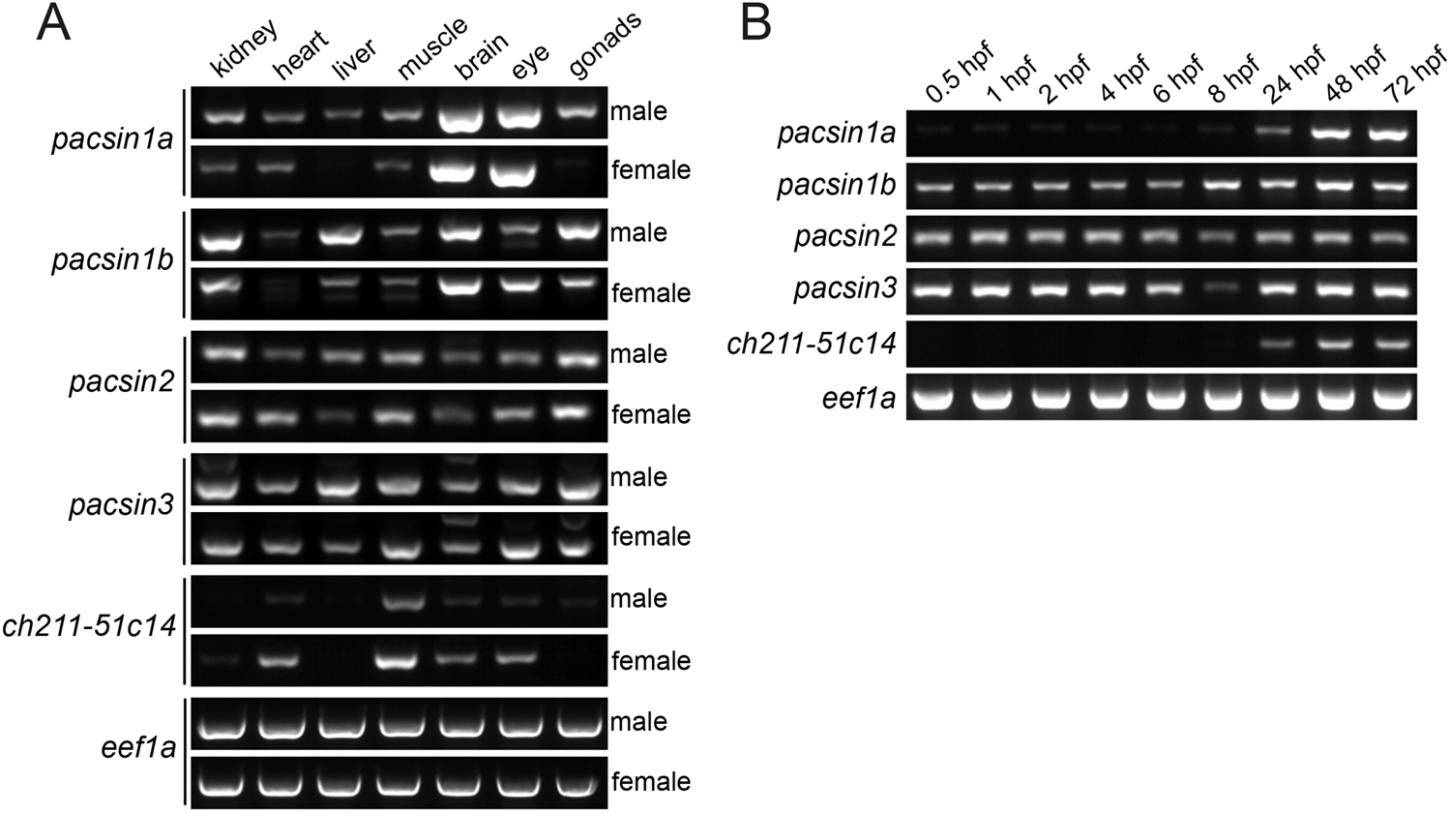
**Tissue and developmental expression of zebrafish *pacsin* genes.** (A) The expression of *pacsin1a*, *pacsin1b*, *pacsin2*, *pacsin3*, and *ch211-51c14-1* mRNA was analysed by RT-PCR in whole organs of male (top panels) and female (bottom panels) adult zebrafish. The housekeeping gene eef1a was used as a positive control. (B) Developmental expression profile of the indicated *pacsin* genes at different developmental timepoints was assessed by RT-PCR. *eef1a* was used as a loading control.

We next performed expression analysis of the zebrafish pacsins. *pacsin1a* is predominantly expressed in the nervous system (Fig. 1A), which is similar to mammalian *PACSIN1* (Kessels and Qualmann, 2004). In contrast, *pacsin1b* is more widely expressed, although the brain is again one of the most highly expressing tissues. *pacsin2* is expressed ubiquitously across all tissues (Fig. 1A), as is the case for the mammalian orthologue (Kessels and Qualmann, 2004). *pacsin3* is also expressed ubiquitously (Fig. 1A), in contrast to the mammalian orthologue, which is predominantly found in heart and skeletal muscle (Kessels and Qualmann, 2004). Interestingly, the *pacsin3* paralogue *ch211-51c14.1* shows this enrichment in the heart and skeletal muscle (Fig. 1A), similar to the mammalian *PACSIN3*, supporting the view it is a functional gene product in zebrafish. We did not assess the expression profile of *zgc:91999* as it was not fully annotated at the time of the analysis. Analysis of the developmental expression of the zebrafish pacsins revealed that *pacsin1b*, *pacsin2* and *pacsin3* are expressed throughout embryonic development (Fig. 1B). All three transcripts are present as maternal pools and their expression is maintained at a relatively constant level through to 72 h post-fertilisation. In contrast, *pacsin1a* and *ch211-51c14.1* are not expressed before 1 day post-fertilisation (dpf), and expression increases beyond this time (Fig. 1B). Expression of these two genes therefore coincides with major organogenesis events, consistent with their organ-specific expression pattern in adult animals.

### Loss of Pacsin2 in zebrafish does not affect viability or gross morphology

**Fig. 2.**
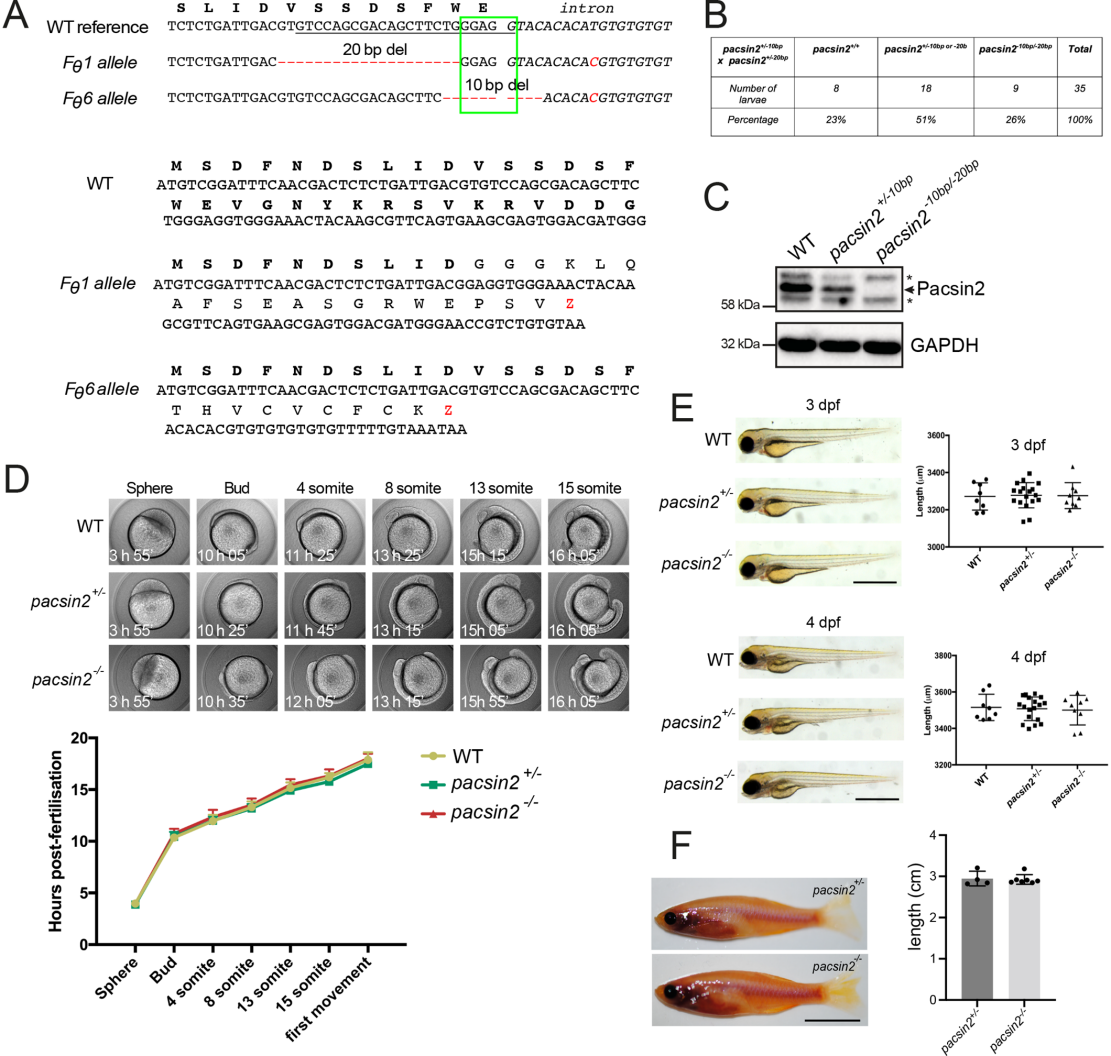
**Generation and normal development of *pacsin2* knockout zebrafish.** (A) Top, mutant *pacsin2* alleles in F0 founder zebrafish (*F*_θ_) aligned to a wild-type reference sequence. Red dashes indicate deleted nucleotides. Red nucleotides indicate an SNP present in the intronic sequence. The green box indicates a splice donor site at the end of exon 2. The underlined sequence is the guide RNA target sequence. Bottom, predicted protein sequences encoded by the two indicated *pacsin2* mutant alleles. (B) Larvae produced by crossing *pacsin2^+/−10bp^* and *pacsin2^+/−20bp^* zebrafish are at the expected Mendelian ratio. (C) Western blot analysis of whole-brain protein lysates from wild-type, *pacsin2^+/−10bp^* and *pacsin2^−10bp/−20bp^* adult fish blotted for Pacsin2 (arrow indicates Pacsin2 band, asterisks indicate cross-reacting proteins) and GAPDH. (D) Wild-type, *pacsin2^+/−^* and *pacsin2^−/−^* embryos were imaged every 10 min over the first 20 h post-fertilisation and developmental progression scored. Data were analysed using two-way ANOVA and Dunnett's multiple comparison test. In all cases *P*>0.05, *n*=7-10 embryos per genotype. Error bars=s.d. (E) Zebrafish larvae at 3 and 4-dpf were imaged on a brightfield dissecting microscope and assessed for gross morphology (left). Body length was also measured (right). Data were analysed by one-way ANOVA followed by a *post-hoc* Tukey's multiple comparison test. In all cases *P*>0.05. *n*=8-18 larvae per genotype. Error bars=s.d.; scale bar: 1 mm. (F) Left, 5-month-old adult male *pacsin2^+/−^* and *pacsin2^−/−^* clutch-mates were photographed and inspected for gross morphology (left). Right, body axis length was measured and analysed using an unpaired *t*-test, *P*>0.05. *n*=4 (*pacsin2^+/−^*) and 7 (*pacsin2^−/−^*)*.* Error bars=s.d.; scale bar: 1 cm.

In this study we wanted to assess the functional importance of Pacsin2 *in vivo*, and therefore generated a stable knockout zebrafish line using CRISPR/Cas9. Guide RNAs targeting exon 2, the first coding exon of *pacsin2*, were used to generate indels, which sequencing confirmed comprised either a 10 bp or 20 bp deletion, and F1 animals created by outcrossing founders containing these mutations with wild-type fish. Both mutations are expected to result in nonsense mutation and should they be expressed, a severely truncated and non-functional protein (Fig. 2A). We failed to obtain breeding pairs containing the same *pacsin2* mutation due to inherent sex bias during breeding in the aquarium at the time the lines were generated, which resulted in a lack of females containing homozygous *pacsin2* mutations. The sex bias was independent of genotype (the same sex ratios were observed with wild type and other lines at that time) and we attribute it to environmental conditions. Compound heterozygotes containing both mutant alleles were therefore generated and used for subsequent experiments. The mutations were present in offspring at an expected Mendelian ratio, indicating that loss of *pacsin2* does not affect embryonic viability (Fig. 2B). This is supported by our ability to subsequently generate homozygous *pacsin2^−10bp/−10bp^* mutants that are viable (Fig. S2). This finding also argues against complementarity between the two mutant alleles. Western blotting of whole-brain lysates of juvenile animals with an antibody raised to zebrafish Pacsin2 confirmed the loss of protein in the compound heterozygote, showing that both mutant alleles fail to generate Pacsin2 protein (Fig. 2C). Functional experiments were performed on *pacsin2* null animals created by crossing compound heterozygote males (*pacsin2^−20bp/−10bp^*) with females harbouring the 10 bp or 20 bp deletion (*pacsin2^wt/−20bp^* ^or *wt/−10bp*^), or by crossing male *pacsin2^+/−10bp^* fish with female *pacsin2^+/−20bp^* fish. For simplicity, *pacsin2^+/−10bp^* or *pacsin2^+/ −20bp^* larvae will hereon in be referred to as *pacsin2^+/−^* as we did not distinguish between mutant alleles in our experiments. Likewise, as we have shown that *pacsin2^−10bp/−20bp^* fish have no Pacsin2 protein, we will refer to these as *pacsin2^−/−^* for simplicity.

Because *pascin2* is expressed throughout embryogenesis, we assessed whether loss of the protein would affect embryonic development and morphogenesis. Time-lapse imaging of *pacsin2^−/−^* embryos generated from a heterozygote in-cross indicated no significant developmental delay up to 16 h post-fertilisation (hpf), assessed using gross morphology (Fig. 2D). Analysis of *pacsin2^−/−^* larvae at 3 and 4 dpf also indicated no developmental delay, and no difference in larval morphology or size (Fig. 2E). Although we cannot exclude the possibility of maternal transcript contributing to embryonic survival, morpholino data suggest this is unlikely. Both translation blocking (E2) and splice-blocking (E4) morpholinos targeting *pacsin2* transcripts, when injected into one cell stage embryos, did not affect larval development or viability (Fig. S3A-D). Although higher levels of the morpholinos did have some effect on viability and larval morphology, this was likely due to off-target effects at these higher doses as it did not correlate with the level of Pacsin2 knock-down (Fig. S3A-D). Analysis of *pacsin2^−/−^* mutant zebrafish at juvenile and adult stages indicated normal morphology and size (Fig. 2F). Thus, loss of Pacsin2 does not affect zebrafish development, morphogenesis or viability.

### Loss of Pacsin2 causes a proximal tubular uptake defect

**Fig. 3.**
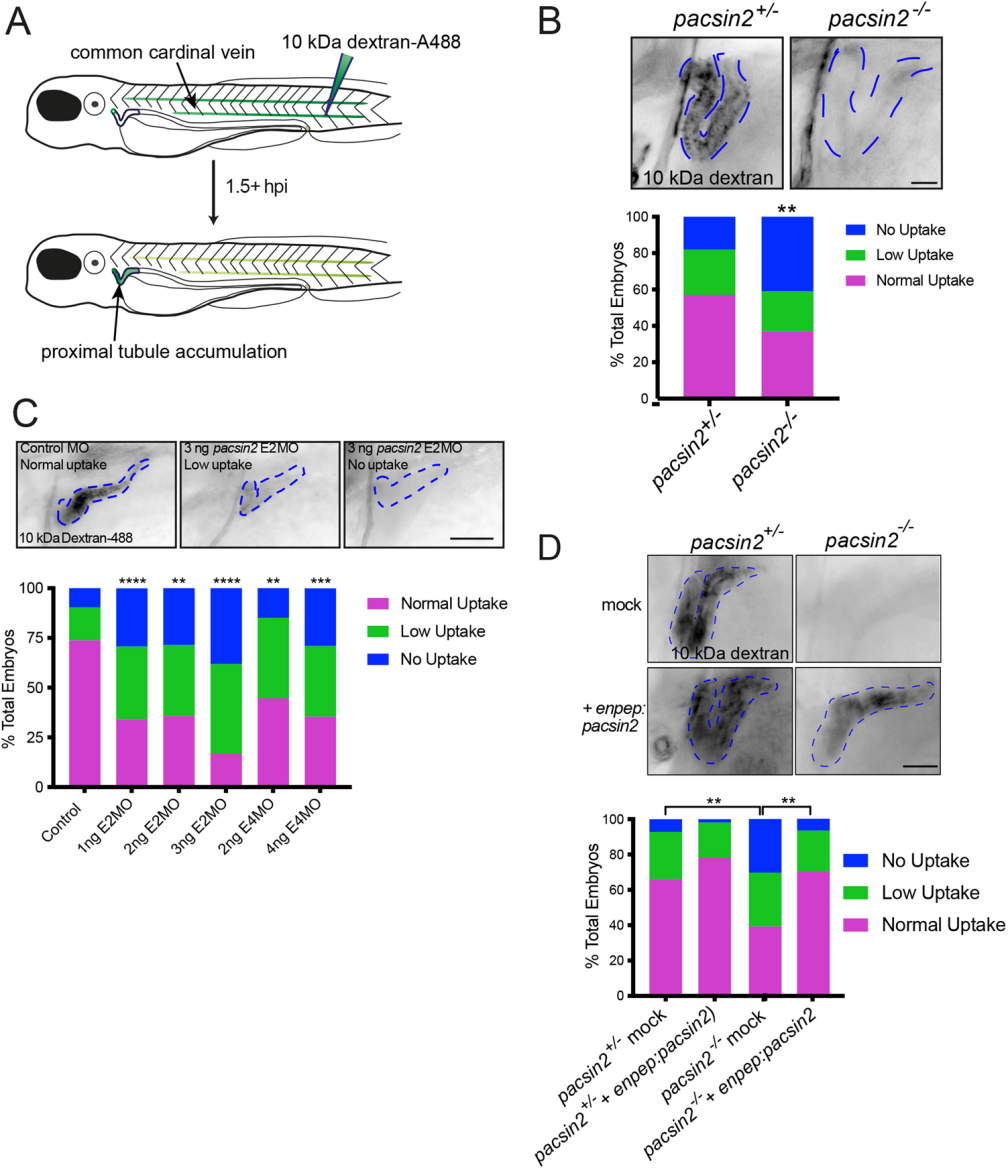
**Depletion or knockout of Pacsin2 in zebrafish larvae impairs renal tubule endocytosis.** (A) Schematic representation of dextran reabsorption assay performed on zebrafish larvae. (B) Top, representative image of 4 dpf *pacsin2^+/−^* (left) and *pacsin2^−/−^* (right) larvae 1.5 h post-injection with 10 kDa dextran-A488, displaying normal and no uptake phenotypes, respectively. Scale bar: 25 µm. The blue dashed line indicates the position of the proximal tubule. Bottom, the percentage of larvae showing normal, low or no uptake phenotypes in *pacsin 2^+/−^* and *pacsin 2^−/−^* larvae. Data were analysed using a Chi-squared test. **P<0.01. *n*=67 (*pacsin2^+/−^*) and 41 (*pacsin2^−/−^*). (C) Top, representative images of 3 dpf control larvae showing normal accumulation of 10 kDa dextran-A488 at 1.5 h post-injection and morphant embryos displaying low or no uptake phenotypes, respectively. The blue dashed lines outline the position of the proximal tubule. Scale bar: 50 µm. Bottom, quantification of uptake phenotypes of control, E2MO and E4MO morphant larvae. Data were analysed using a Chi-squared test. **P<0.01, ***P<0.001, ****P<0.0001. *n*=28-84 larvae per genotype. (D) Top, representative images of *cmlc:GFP* (top row, mock) and *enpep:pacsin2* (bottom row) rescued *pacsin2^+/−^* and *pacsin2^−/−^* larvae. The blue dashed lines outline the proximal tubule. Scale bar: 60 µm. Bottom, percentage of total larvae scored for uptake phenotypes. Data were analysed using a Chi-squared test. **P<0.01. *n*=23-56 larvae per genotype.

We next wanted to assess whether loss of Pacsin2 affected endocytosis in the zebrafish renal tubule. Megalin-dependent retrieval of low molecular weight proteins is an extremely active process within the proximal tubule, reliant on a high rate of endocytic uptake and receptor recycling (Christensen et al., 2012). Considering the endocytic roles described for mammalian PACSIN2, we hypothesised that it may be required for proximal tubular endocytosis. To test this possibility, we used a previously described assay whereby fluorescent low molecular weight dextran is injected into the bloodstream of zebrafish larvae, and its filtration by the glomerulus and subsequent uptake into the pronephros (larval kidney tubule) assessed using fluorescence microscopy (Christou-Savina et al., 2015; Oltrabella et al., 2015) (Fig. 3A). As shown in Fig. 3B, categorical scoring of dextran fluorescence in the pronephros revealed reduced uptake in *pacsin2*-knockout larvae compared to heterozygote controls. This was not due to any defect in tubular morphogenesis as this was normal upon the loss of Pacsin2 (Fig. S4A). Nor was it due to effects upon glomerular filtration, indicated by the clearance of dextran from the bloodstream, as this too was normal in the *pacsin2*-knockout larvae (Fig. S4B). *pacsin2* morphants also showed reduced dextran uptake (Fig. 3C), indicating the phenotype is specific. This was further indicated by rescue of dextran uptake in the knockout animals upon re-expression of wild-type Pacsin2 selectively in the pronephros (Fig. 3D), which was achieved using the *enpep* promoter (Seiler and Pack, 2011). These results indicate a requirement for Pacsin2 in proximal tubular endocytosis.

### Loss of Pacsin2 causes a reduction in abundance of apical endocytic compartments

**Fig. 4.**
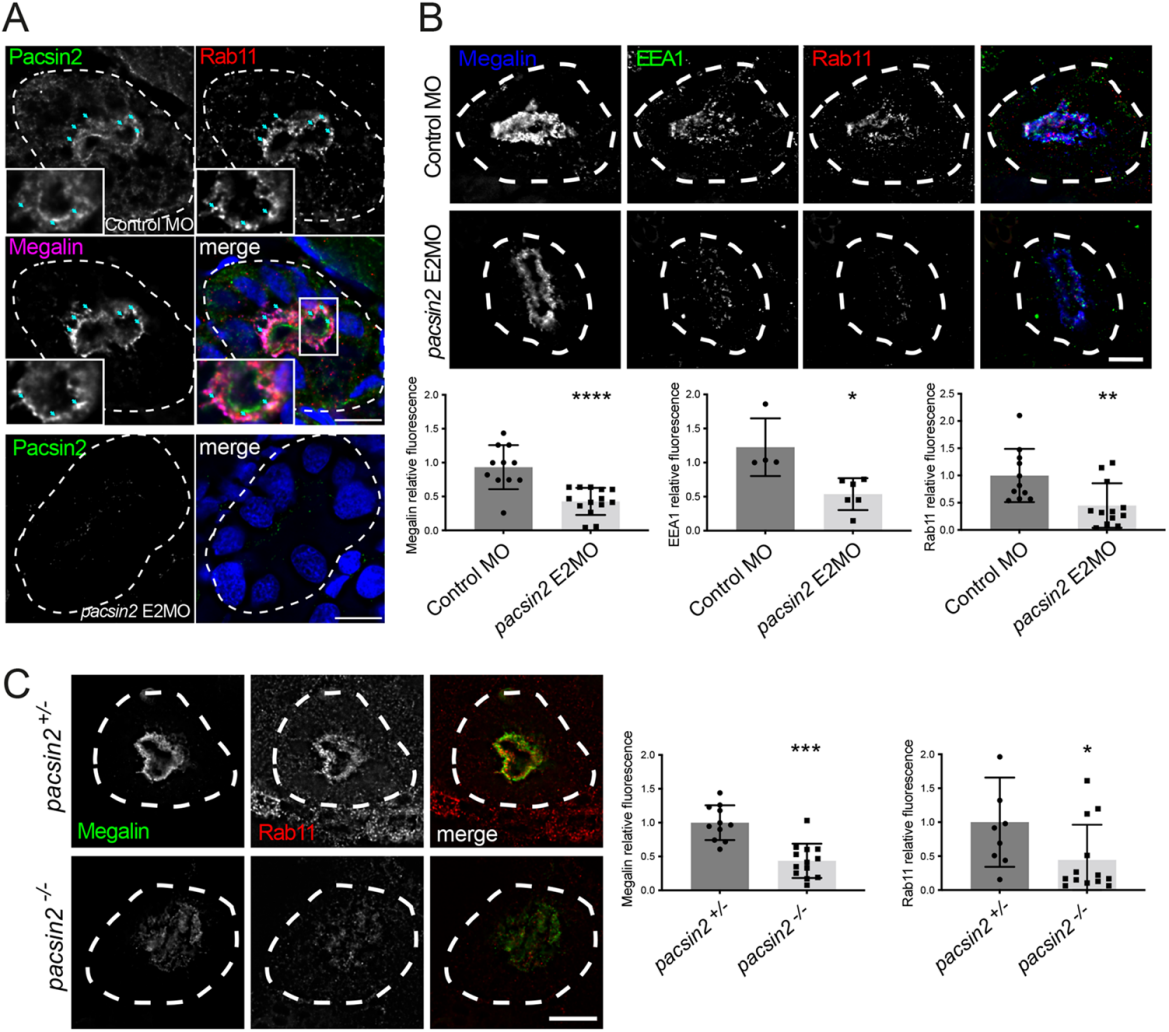
**Loss of Pacsin2 causes reduced abundance of megalin and endocytic markers in the proximal tubule.** (A) Top, transverse cryosections of proximal tubule were immunolabeled for Pacsin2, Rab11 and megalin in 3 dpf wild-type larvae. Insets depict an enlarged view of the boxed region, rotated 90˚ clockwise. Blue arrows indicate regions of colocalisation between Pacsin2, Rab11 and megalin. Bottom, 3 dpf *pacsin2* E2MO morphant larvae labelled with anti-Pacsin2 antibody. (B) Top, transverse cryosections through the pronephros of 3 dpf control morphant or *pacsin2* E2MO morphant larvae immunolabeled for megalin, EEA1 and Rab11. Bottom, quantification of relative signal intensity of megalin, EEA1 and Rab11, respectively, between control and *pacsin2* morphants. *n*=5-12 for control morphants and 6-14 for *pacsin2* morphants. Unpaired *t*-test, * *P*<0.05, ** *P*<0.01, **** *P*<0.0001. Error bars=s.d. (C) Left, transverse sections through the proximal tubule of 4 dpf *pacsin2^+/−^* or *pacsin2^−/−^* larvae immunolabelled for megalin and Rab11. Right, quantification of relative signal intensity of megalin and Rab11, respectively, between *pacsin2^+/−^* and *pacsin2^−/−^* larvae. *n*=11 and 13 for megalin, and 9 and 13 for Rab11 for *pacsin2^+/−^* and *pacsin2^−/−^*, respectively. Unpaired *t*-test, *P<0.05 *** *P*<0.001. Error bars=s.d. Dashed white lines indicate the outer margin of the pronephros. Scale bars: 10 µm.

We next wanted to assess whether loss of Pacsin2 would affect the abundance and morphology of endocytic compartments in the proximal tubule. Active endocytosis in renal proximal tubular cells occurs at the apical membrane, and consequently there is a concentration of endocytic compartments and machinery at the apical pole of the cell. Immunofluorescence microscopy of endogenous Pacsin2 showed enrichment at the apical membrane of proximal tubule cells (Fig. 4A), consistent with a previous study showing apical enrichment of Pacsin2 in the mouse renal tubule (Yao et al., 2013). Pacsin2 could also be visualised in sub-apical puncta that showed partial co-localisation with megalin, which populates the apical endosomal system, and Rab11, which marks apical vacuolar endosomes in proximal tubule cells (Mattila et al., 2014) (Fig. 4A; Fig. S5). This suggests that Pacsin2 can also reside on sub-apical endosomal compartments. The specificity of the Pacsin2 labelling was confirmed using morphant larvae (Fig. 4A). To assess the effects of Pacsin2 loss upon the endosomal network, *pacsin2* morphants were labelled for Rab11 and EEA1, which is a marker of early endosomes, as well as megalin. There was a reduction in the intensity of both endosomal markers at the apical region of proximal tubular cells, suggesting reduced abundance of endocytic compartments (Fig. 4B). The megalin signal was also reduced (Fig. 4B), possibly due to changes in its trafficking within the endosomal system, which could result in excessive shedding at the apical pole or degradation in lysosomes (Fatah et al., 2018; Gena et al., 2010). A similar reduction in Rab11 and megalin abundance was also seen in the *pacsin2*-knockout larvae (Fig. 4C), supporting the view that loss of Pacsin2 causes loss of apical endosomal compartments and megalin in the proximal tubule.

**Fig. 5.**
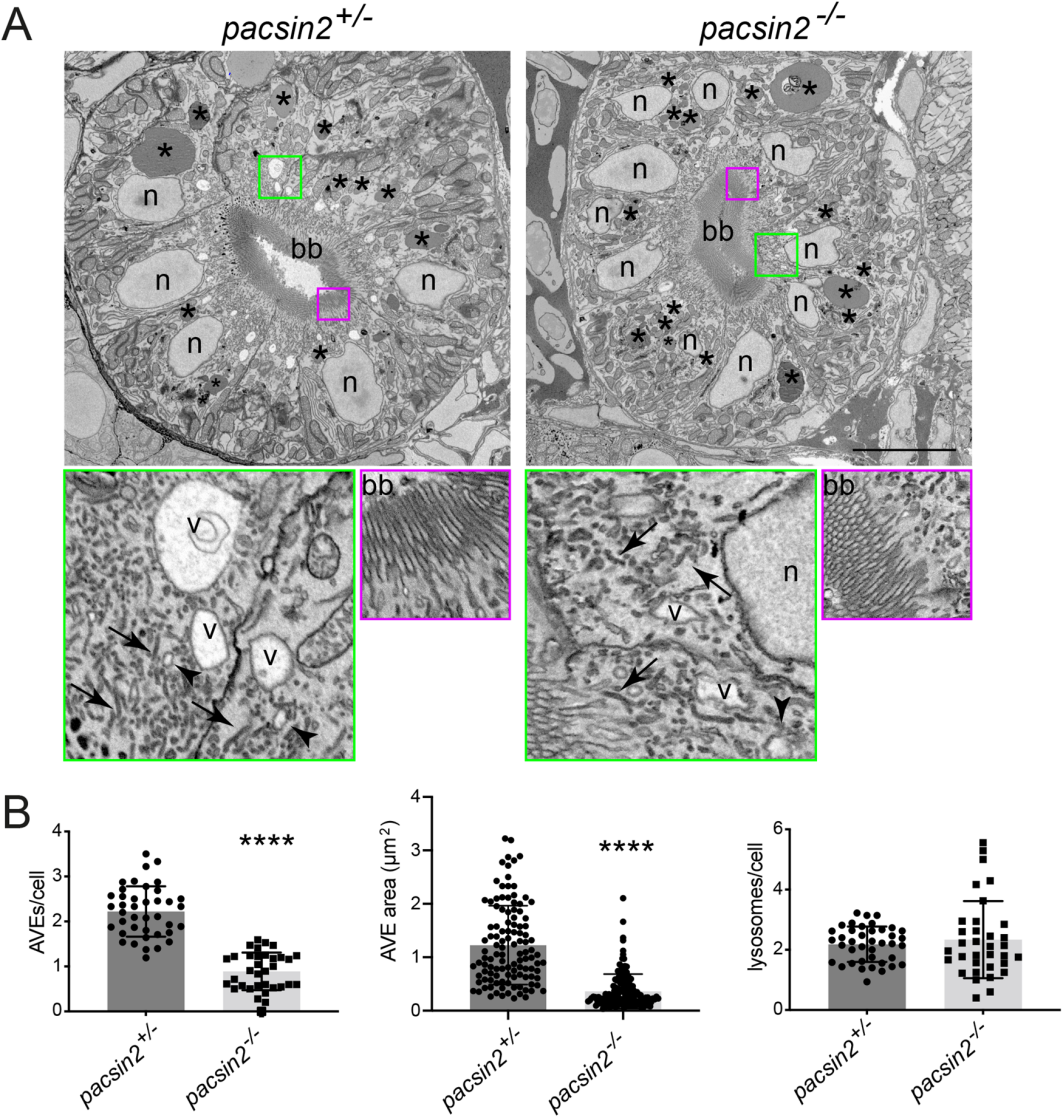
**Loss of Pacsin2 results in reduced abundance of the apical endocytic organelles in the proximal tubule.** (A) Block-face SEM images of transverse proximal tubule sections from *pacsin2^+/−^* (left panels) and *pacsin2^−/−^* (right panels) 4 dpf larvae. Top panels show whole cross-section of the proximal tubule, bottom panels show enlarged boxed regions indicated in green and red. Top panels: scale bar: 10 µm. Green and red boxed areas=4 µm and 3 µm, respectively. bb, brush border; V, apical vacuolar endosome; *N*, nucleus; *, lysosomes; arrows, dense apical recycling tubules; arrowheads, endocytic vesicles. (B,C) Average number and size of AVEs, respectively, and (D) number of lysosomes per cell. Unpaired *t*-test, **** *P*<0.0001. For B and D, *n*=40 (*pacsin2^+/−^*) and 34 (*pacsin2^−/−^*). For C, *n*=114 (*pacsin2^+/−^*) and 136 (*pacsin2^−/−^*). Error bars=s.d.

To more directly assess the abundance and morphology of endocytic compartments we used block face scanning electron microscopy, similar to how it was previously performed (Oltrabella et al., 2021, 2015). The endocytic structures are readily identifiable using this approach, with the apical vacuolar endosomes, which serve as a hybrid sorting and recycling compartment, seen as electron lucent structures close to the numerous electron dense recycling tubules found in the sub-apical region (Fig. 5A). The overall morphology and polarisation of the proximal tubular cells was normal, with a clear apical brush border present in both control and *pacsin2*-knockout larvae (Fig. 5A). Strikingly though, there was a reduction in number and overall area occupied by the apical vacuolar endosomes (AVE) in the *pacsin2* knockout (Fig. 5A,B). There also appeared to be fewer recycling tubules, but we were not able to reliably quantify this effect. In contrast to endosomes, the lysosomes of proximal tubule cells appeared unaffected in the *pacsin2* knockout (Fig. 5A,B). Together, our results indicate a reduction of apical endosomal compartments upon the loss of Pacsin2.

## DISCUSSION

PACSIN2 participates in a number of cellular processes including endocytic traffic, caveolae formation, and ciliogenesis (Quan and Robinson, 2013). The extent to which PACSIN2 contributes to these processes *in vivo*, within different tissues, remains poorly defined. In this study, we report that zebrafish Pacsin2 is required for endocytosis *in vivo*, in the renal tubule. Loss of Pacsin2 causes a deficit in endocytic uptake into the apical pole of renal tubular cells, with a reduction in the abundance of endocytic compartments in these cells. Endocytosis in renal tubular cells is a highly active process, important for clearance of low molecular weight proteins from the renal filtrate, and various studies have shown the importance of different proteins in this process (reviewed in Christensen et al., 2012). Other studies have shown that PACSIN2 can function in both endocytic uptake and recycling in mammalian cells (reviewed in Quan and Robinson, 2013), and both processes may be impaired in our *pacsin2* knockout and morphant larvae. The decrease in abundance of apical endocytic compartments in our experiments suggests endocytic uptake is likely to be affected. A similar albeit more dramatic loss of endocytic organelles is seen when the major endocytic receptor megalin is absent from the proximal tubule (Anzenberger et al., 2006; Kur et al., 2011). We may therefore expect less endocytic uptake and flux through the pathway due to loss of Pacsin2 to result in reduced abundance of endocytic organelles. A role in caveolar uptake is unlikely considering the caveolin proteins are not expressed in the proximal tubule *in vivo* (Zhuang et al., 2011), consistent with a more likely role for Pacsin2 in clathrin-dependent endocytosis. Of note, a recent study has shown that mutation of the PACSIN2 binding partner EHD1, which is involved in endocytic recycling (Naslavsky and Caplan, 2011), causes a rare tubular disorder characterised by low molecular weight proteinuria due to defective endocytic traffic (Issler et al., 2022). Thus, it remains possible that Pacsin2 contributes to both endocytic uptake and recycling within the proximal tubule.

The loss of Pacsin2 in the zebrafish renal tubule did not affect development of this tissue, nor cell polarity or formation and maintenance of the brush border of the proximal tubular epithelial cells. The latter phenotype differs from that seen in the intestine of *PACSIN2*-knockout mice, which have reduced numbers of microvilli and altered microvillar ultrastructure (Postema et al., 2019). This may reflect different requirements for the protein in the renal tubule epithelium compared to the intestinal epithelium, or a species-dependent difference. It will be interesting to analyse the renal tubular epithelium of the *PACSIN2* knockout mouse and also look at other tissues in Pacsin2-deficient zebrafish to assess the extent of *pacsin2* null phenotypes in the two model organisms. Although *pacsin2* is broadly expressed at the tissue level and throughout early development, its loss in both zebrafish (this study) and mouse (Malinova et al., 2021; Postema et al., 2019; Semmler et al., 2018) does not lead to gross changes in morphology or viability. This suggests that PACSIN2 may play only an accessory role in the various processes it participates in, at least in an *in vivo* context, or that there is redundancy or functional compensation by other proteins. Candidates here are other F-BAR proteins, including other members of the PACSIN family, that could replace PACSIN2 function when it is absent. Functional redundancy between zebrafish Pacsin1b and Pacsin2 has previously been shown during ciliogenesis, which interestingly differed in the two tissues analysed (otic vesicle and olfactory placode) (Insinna et al., 2019). Further analysis of functional redundancy between the PACSINs and/or other F-BAR proteins, as well as possible compensatory mechanisms for loss of PACSIN2, should prove informative in this regard.

A previous study showed changes in PACSIN2 expression within the mouse kidney during development and following injury, where it was more highly expressed than in the adult organ at steady state, consistent with a role in nephrogenesis (Yao et al., 2013). This was supported by analysis of kidney tubulogenesis *in vitro*, which was impaired upon PACSIN2 knockdown. Our data indicate that Pacsin2 is dispensable for kidney development *in vivo* in zebrafish, and the viability of *PACSIN2* knockout mice also suggests a minor if any defect in formation of the kidneys in this model. Our data rather support a role for Pacsin2 in the kidney post-development, in maintaining optimal endocytosis within the proximal tubule. Single cell transcriptomic analysis of the mouse kidney shows that *PACSIN2* is expressed throughout the adult renal tubule (Park et al., 2018), consistent with a function in maintaining renal physiology. It is the most abundant of the three mammalian PACSINs in all segments, including the proximal tubule, as would be expected if it were to function in endocytosis at this location.

## MATERIALS AND METHODS

### Antibodies

Polyclonal antibodies to zebrafish Pacsin2 were generated in sheep by immunising with a recombinant GST-tagged Pacsin2 construct encoding amino acids 301-388. Immunisation and serum collection were by Orygen Antibodies Ltd. Antibodies were affinity purified from serum by first clearing on GST beads alone followed by affinity purification on the GST-Pacsin2 recombinant protein. Polyclonal antibodies to zebrafish megalin were generated in rabbits against a GST fusion to the cytoplasmic domain, and affinity purified on the recombinant protein. Also used in this study were goat anti-EEA1 (Santa Cruz Biotechnology, sc-6415), mouse anti-Rab11 (BD Transduction Labs, 610657), mouse 3G8 anti-proximal tubule (European Xenopus Resource Centre, Portsmouth, UK), and mouse anti-GAPDH (Santa Cruz Biotechnology, sc-25778). Fluorophore-and HRP-conjugated secondary antibodies were purchased from Thermo Fisher Scientific.

### Molecular biology

All constructs were made using standard molecular biology techniques. Zebrafish *pascin2* gene sequences is designated on Ensembl as ENSDARG00000078014. Full-length zebrafish *pacsin2* cDNA was cloned into pT2KXIGDin-enpep vector (Dr Michael Pack, University of Pennsylvania, USA) for expression in zebrafish pronephric tubules. GST-tagged *pacsin2* (encoding amino acids 301-388) was cloned into pFAT2 for bacterial expression and antibody production and purification. cDNA encoding the cytoplasmic domain of zebrafish megalin (amino acids 4464-4673) was cloned into pGEX-4T for antibody production and purification. Primer sequences for all manipulations are available upon request. All constructs were verified by DNA sequencing. Plasmid encoding GFP under control of the cardiomyosin light chain 2 promoter (*cmlc2*:GFP) was obtained from Dr Adam Hurlstone (University of Manchester, UK).

### Zebrafish strains and husbandry

Zebrafish were raised and maintained at the University of Manchester Biological Services Unit according to the UK Animals (Scientific Procedures) Act 1986. The AB strain was used for morpholino studies and the non-pigmented Casper (White et al., 2008) strain was used to generate *pacsin2* mutants. AB embryos used in experiments were transferred at 2 hpf to chorion water +0.003% phenylthiourea (PTU) to prevent pigment development. Generation of the *pacsin2* mutant line was performed with CRISPR/Cas9, and this line was maintained as heterozygotes.

### RNA isolation and PCR

Total RNA was isolated from zebrafish embryos or adult tissues using Trizol (Invitrogen) and reverse-transcribed with Superscript First Strand (Invitrogen) to produce cDNA. For analysis of amplification products, cDNA was amplified using standard PCR conditions and appropriate primer pairs. For PCR of genomic DNA, genomic DNA was extracted from single or pooled embryos or was isolated from fin clips (1 mm^2^) taken from juvenile fish by extraction into 50 mM NaOH, heating to 95°C, neutralisation using Tris, pH 8, and centrifugation to remove insoluble debris. PCR was performed using standard conditions and appropriate primer pairs.

### RNA, DNA and morpholino injections in zebrafish

For CRISPR/Cas9 mutagenesis, guide RNA targeting exon 2 of zebrafish *pacsin2* (GTCCAGCGACAGCTTCTGGG) was co-injected with Cas9 mRNA and protein (40 ng/µl sgRNA, 100 ng/µl Cas9 mRNA, 300 ng/µl EnGen Cas9 protein (NEB); injected at 1 nl volume) into one-cell-stage embryos. Mutagenesis was assessed by performing PCR of the genomic locus followed by restriction digestion using AlwNI, which cuts at the guide target sequence. Mutagenesis confers resistance to digestion. This method was also used for routine genotyping of zebrafish. Discrimination between the two mutant *pacsin2* alleles (*–10 bp* and *−20 bp*) was done by DNA sequencing. For rescue experiments, capped mRNA encoding tol2 transposase was transcribed from the pCS2-FA vector (Dr Michael Pack, University of Pennsylvania, USA) using the mMessage mMachine kit (Ambion) and approximately 1 nl of a mix of 10 ng/µl pT2KXIGDin-*enpep* vector containing *pacsin2* coding sequence, 10 ng/µl *cmcl2*:GFP vector and 20 ng/μl tol2 transposase mRNA was injected into one-cell-stage embryos. Morpholinos were obtained from GeneTools. Control morpholino was described previously (Ramirez et al., 2012); 1-3 nl of morpholino targeting zebrafish *pacsin2* (E2, ATGTCTGAAAGAACAACAGCACAGA; E4, CTCGCGCTGCCTGTGTTTACCTCCT) was injected into the yolk sac of one-cell-stage embryos.

### Injection and analysis of endocytic tracers

Lysine-fixable Texas Red- or Alexa 488-conjugated 3 kDa or 10 kDa dextran (Thermo Fisher Scientific) was prepared in PBS at 2 µg/µl final concentration. The injected volume was adjusted individually for each tracer used based on the total fluorescence in the larvae circulatory system. Zebrafish larvae at 72 hpf were anesthetised with 0.2 mg/ml MS222 (Sigma-Aldrich), in chorion water, and tracer injected into the common cardinal vein using a glass micropipette PLI-90 Pico-Injector (Harvard Apparatus). Pronephric uptake was assessed at between 1-2.5 h after injection on whole mounts using a fluorescence dissecting stereomicroscope (Leica MZ10F).

### Timelapse imaging of zebrafish embryos

Embryos were mounted in 1% low melting point agarose, overlaid with chorion, warmed to 28°C and brightfield images acquired every 10 min on an Eclipse Ti inverted microscope (Nikon) using 10× Plan Fluor objective, the Nikon filter set for GFP and a pE-300 LED (CoolLED) fluorescent light source. NIS Elements AR.46.00.0 software (Nikon) was used to allow multiple embryos to be imaged every 10 min over the course of 24 h, with automatic refocusing. A Retiga R6 (Q-imaging) camera was used to capture single plane images. Images were analysed using NIS Elements Viewer (Nikon) software.

### Fluorescence microscopy

Zebrafish larvae were fixed overnight using 4% PFA. For cryosectioning, larvae were mounted in cryosectioning moulds, frozen on dry ice and sectioned using a Leica CM3050 S cryotome. Cryosections were rehydrated with PBS for 5 min at room temperature and blocked overnight at 4°C in PBS containing 0.1% Triton and 5% Donkey serum. Incubation with primary antibodies in blocking solution was for 4 h at room temperature or overnight at 4°C, followed by secondary antibodies in blocking solution for 4 h at room temperature. Samples were mounted on coverslips in Mowiol. Images were captured with an Olympus IX83 inverted microscope using Lumencor LED excitation, a 100×/1.35 UPlanFl objective and the Penta filter set (Chroma). Images were collected using a R6 (Qimaging) CCD camera. Images were acquired using Metamorph v7.10.09.119 (Molecular Devices). Z-stacks of cryosections were acquired with a Z optical spacing of 0.2 μm and raw images deconvolved using the Huygens Pro software (SVI). For whole mount immunolabelling, fixed larvae were dehydrated in 100% methanol at −20°C overnight, rehydrated at room temperature and washed PBS containing 0.1% Tween-20. Proteinase K treatment (10 µg/ml, Sigma-Aldrich) was performed for 5 min, and larvae were blocked overnight at 4°C in PBS containing 0.1% Tween-20 and 10% FCS. Samples were labelled as for cryosections and mounted by overlaying with 1% low melting point agarose. Samples were imaged using a Leica TCS SP5 AOBS inverted confocal microscope using a 20× HC PL Fluotar (PH2) objective with 0.75× confocal zoom. Three-dimensional optical stacks were acquired using a step size of 1 µm, and images are displayed as maximum intensity projections.

### Block face scanning electron microscopy

Serial block face scanning electron microscopy was performed according to (Oltrabella et al., 2015). Images were analysed using ImageJ. Endocytic and lysosomal compartments were defined by morphology. Vacuolar endosomes are oval or spherical membrane-enclosed compartments of a diameter greater than 500 nm, with an electron sparse lumen that contains varying degrees of granular material. Lysosomes are electron dense oval or spherical membrane-enclosed compartments of a diameter greater than 500 nm.

### Statistical tests

All statistical analyses and graphs were performed with GraphPad Prism version 9. Values are presented as the mean±s.d., and are from a minimum of three independent experiments. The *n* numbers represent sample sizes. The statistical tests used in each case are indicated in the figure legends. All data were first tested for normality. Differences between two independent groups were compared using an unpaired Student's *t*-test. Differences between means of more than two comparison groups were analysed using one-way or two-way ANOVA, with adjustments on multiple comparison tests performed using either the Dunnett, Tukey or Sidak method, depending on the experiment. The Chi-squared test was used to analyse the categorical data obtained in the renal uptake experiments. Survival data were assessed using the non-parametric log-rank (Mantel–Cox) test.

## Supplementary Material

Supplementary information
