# Supplementary material for: Pacsin2 is required for endocytosis in the zebrafish pronephric tubule
Source: Biol Open. 2022 Jun 23;11(6):bio059150. doi: 10.1242/bio.059150 (PMC9235069; doi:10.1242/bio.059150)
Supplement: Supplementary information [file biolopen-11-059150-s1.pdf]

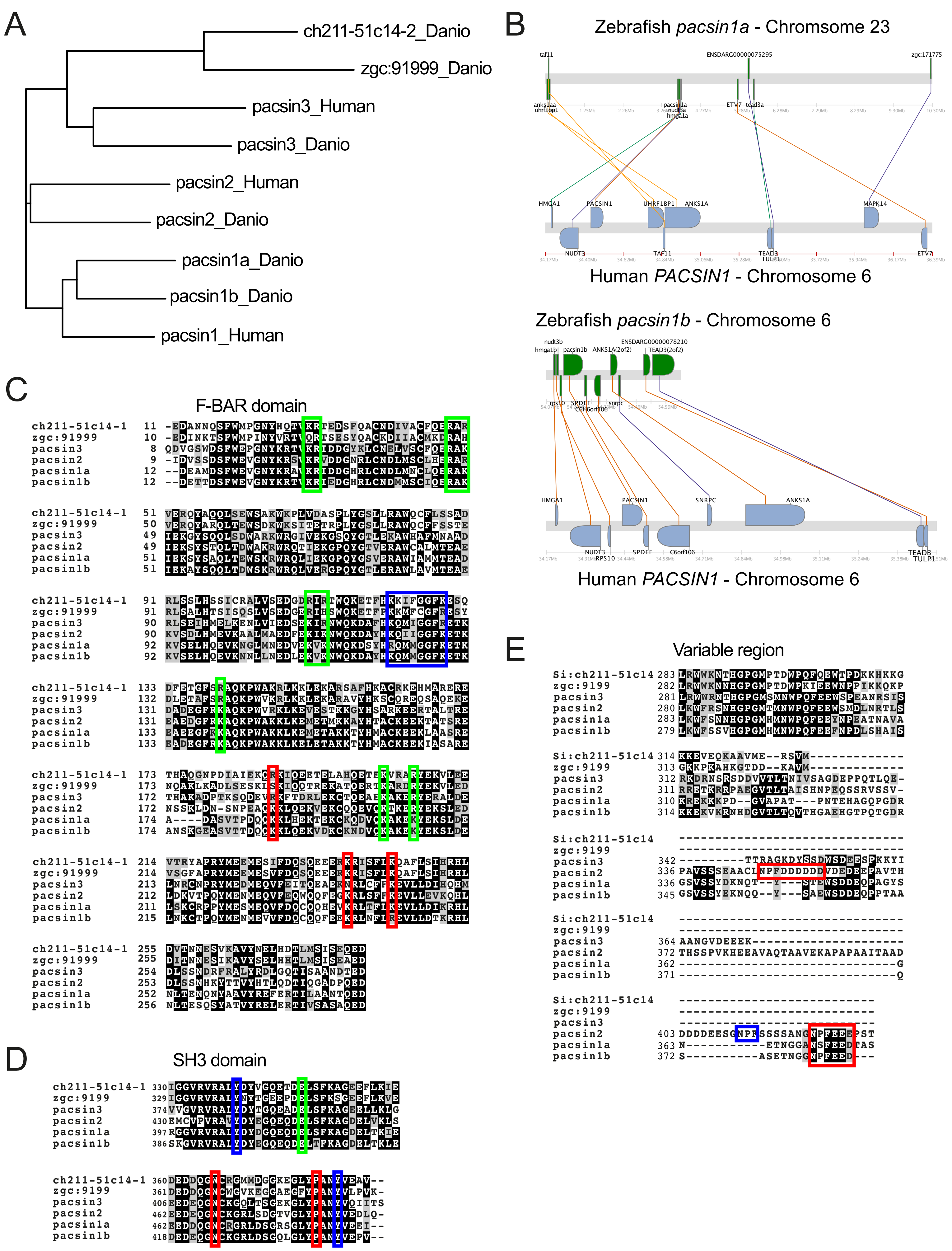

Morgan et al, Fig S1

Biology Open • Supplementary information

Fig. S1. Evolutionary conservation of pacsins in zebrafish. (A) Phylogenic tree from multiple sequence alignments of human and zebrafish *pacsin* family members. **(B)** Synteny traces using a 50-gene window demonstrates high levels of chromosome arrangement conservation between regions in zebrafish housing *pacsin1a* (top) and *pacsin1b* (bottom) compared to chromosome 6 in human containing *PACSIN 1*. **(C-E)** Multiple protein sequence alignment of the F-BAR (C), SH3 domain (D) and variable region (E) across all zebrafish pacsin family members. Green, blue and red boxes in (C) outline regions of conserved basic residues on the concave face important for lipid-binding, conserved pacsin-specific wedge-loop residues, and conserved basic residues on the convex surface important for membrane binding, respectively. Blue and red boxes in (D) highlight conservation of critical residue pairs that form two proline-binding pockets, and the green box indicates conservation of acidic residues in the specificity pocket. The blue box in (E) highlights the minimal NPF motif, and red boxes indicate extended NPF motifs of the form NPF-[D/E]-[D/E]-[D/E].

A

pacsin2$^{+/-10bp}$ x *pacsin2*$^{+/-10bp}$	*pacsin2*$^{+/+}$	*pacsin2*$^{+/-10bp}$	*pacsin2*$^{-/-10bp}$	Total
Number of larvae	16	31	21	68
Percentage	24%	46%	31%	100%

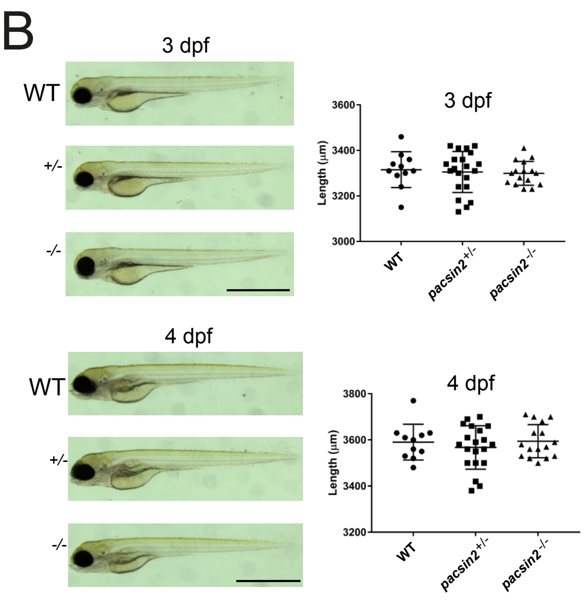

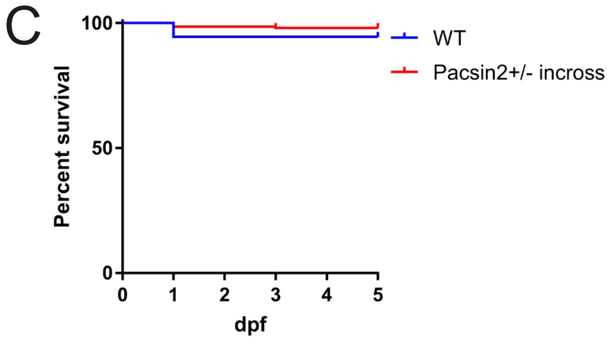

Morgan et al, Fig S2

Fig. S2. Homozygous *pacsin2$^{-10bp/-10bp}$* mutants are viable and have normal morphology. **(A)** Larvae produced by in-crossing *pacsin2*$^{+/-10bp}$ zebrafish are at the expected Mendelian ratio. **(B)** Left, brightfield images of WT, heterozygous *pacsin2*$^{+/-10bp}$ and homozygous *pacsin2*$^{-10bp/-10bp}$ mutants at 3 dpf (top) and 4 dpf (bottom). Right, body lengths of 3 dpf (top) and 4 dpf (bottom) larvae (n =11-16) as indicated. Scale bars, 1 mm. **(C)** Survival of WT, heterozygous *pacsin2*$^{+/-10bp}$ and homozygous *pacsin2*$^{-10bp/-10bp}$ mutants up to 5 dpf (n=11-16).

Biology Open • Supplementary information

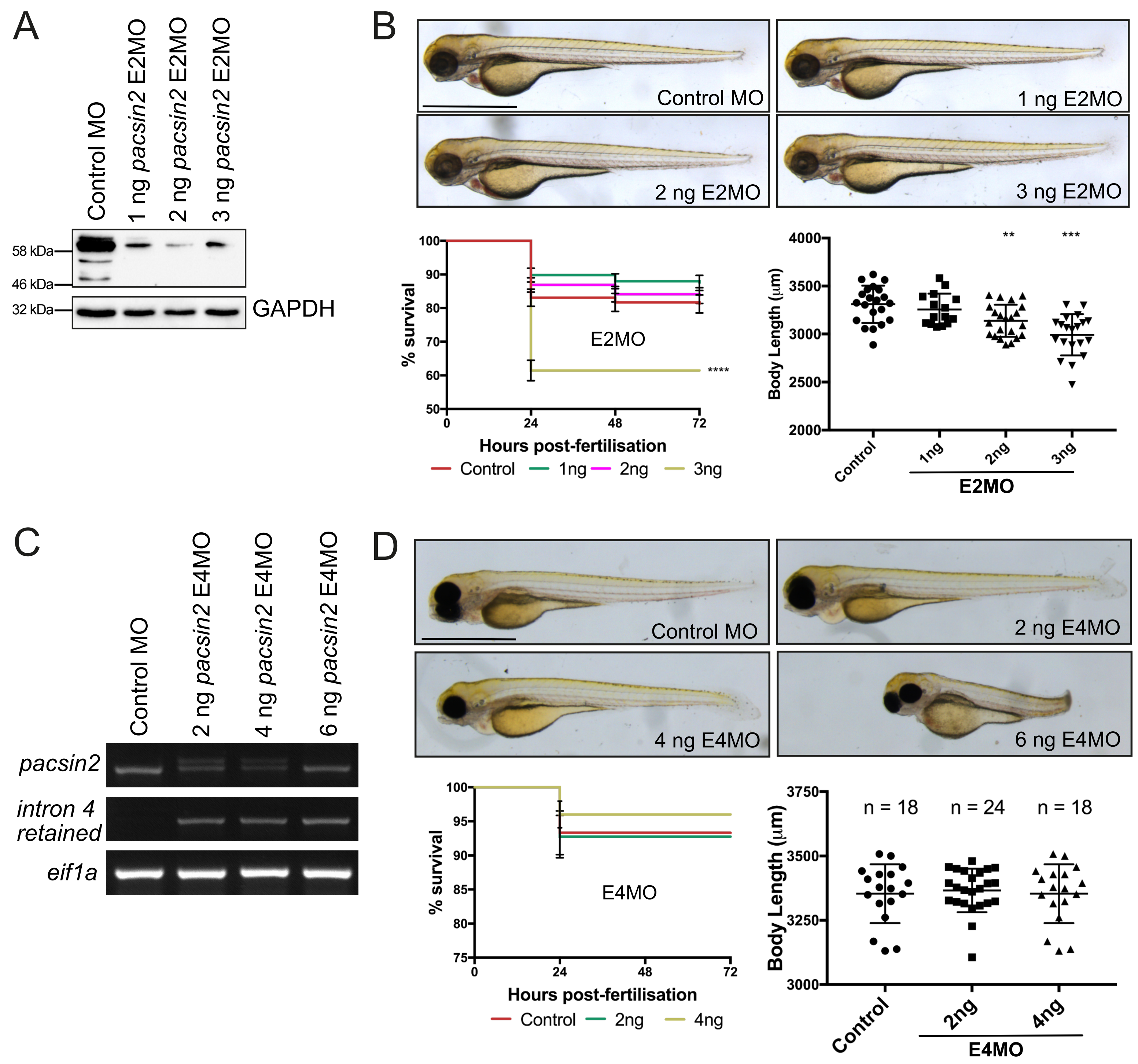

Morgan et al, Fig S3

Fig. S3. *Pacsin2* morphant zebrafish develop and grow normally. (A) 3 dpf larvae injected with 3 ng control morpholino (MO) or 1 – 3 ng pacsin2 E2MO were analyzed by Western blot with antibodies to pacsin2 or GAPDH. **(B)** Top, brightfield images of morphants at 3 dpf. Bottom left, survival of morphants treated with control or increasing doses of *pacsin2* E2MO over 72 hpf. Statistical test used was log-rank (Mantel-Cox) test. **** p<0.0001. n = 213-257 embryos per condition. Bottom right, body length of morphants at 3 dpf larvae. One-way ANOVA followed by Dunnett's multiple comparison was performed. * p=0.04, ** p=0.003. n = 20 larvae per condition. Error bars = SD; scale bar, 1 mm. **(C)** RT-PCR of total mRNA extracted from control or *pacsin2* E4MO morphants injected with 2 ng, 4 ng or 6 ng morpholino. **(D)** Top, brightfield images of morphants at 3 dpf. Bottom left, survival of morphants treated with control or increasing doses of *pacsin2* E4MO over 72 hpf. Statistical test used was log-rank (Mantel-Cox) test. **** p<0.0001. n = 60-100 embryos per condition. Bottom right, body length of morphants at 3 dpf larvae. One-way ANOVA followed by Dunnett's multiple comparison was performed. n = 18-24 larvae per condition. Scale bar, 1 mm.

Biology Open • Supplementary information

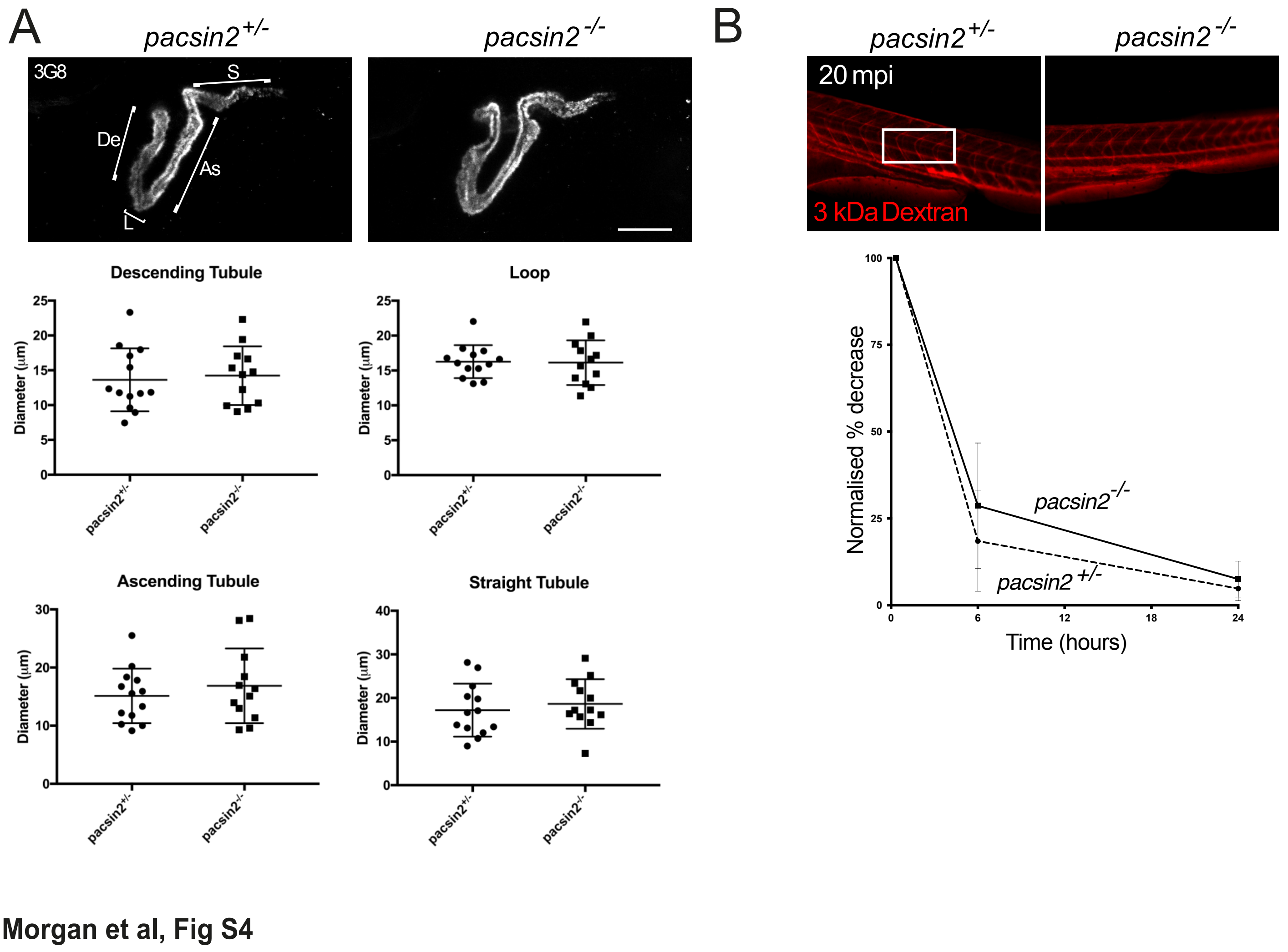

Fig. S4. Loss of pacsin2 does not affect pronephros formation or dye filtration. (A) Top, whole-mount immunofluorescence performed on 4 dpf *pacsin2*$^{+/-}$ and *pacsin2*$^{-/-}$ larvae using 3G8, an apical brush border proximal tubule-specific marker. De = Descending tubule, L = Loop, As = Ascending tubule, S = straight tubule. Scale bar = 50 µm. Bottom, tubule diameters were measured at the widest part of each region marked in (A) and data analyzed using Unpaired t-test, p>0.05 in all cases. n = 13 and 12 larvae for *pacsin2*$^{+/-}$ and *pacsin2*$^{-/-}$, respectively. Error bars = SD. **(B)** Top, *pacsin2*$^{+/-}$ and *pacsin2*$^{-/-}$ larvae were injected simultaneously with 3 kDa dextran (red) and imaged 20 minutes post-injection (mpi) to assess successful injections. Bottom, images were taken at 20 mpi, 6 hpi and 24 hpi and fluorescence measured in the circulatory system. Two-way ANOVA followed by Sidak's multiple comparisons test, p>0.05. n = 5 and 9 larvae for *pacsin2*$^{+/-}$ and *pacsin2*$^{-/-}$, respectively.

Biology Open • Supplementary information

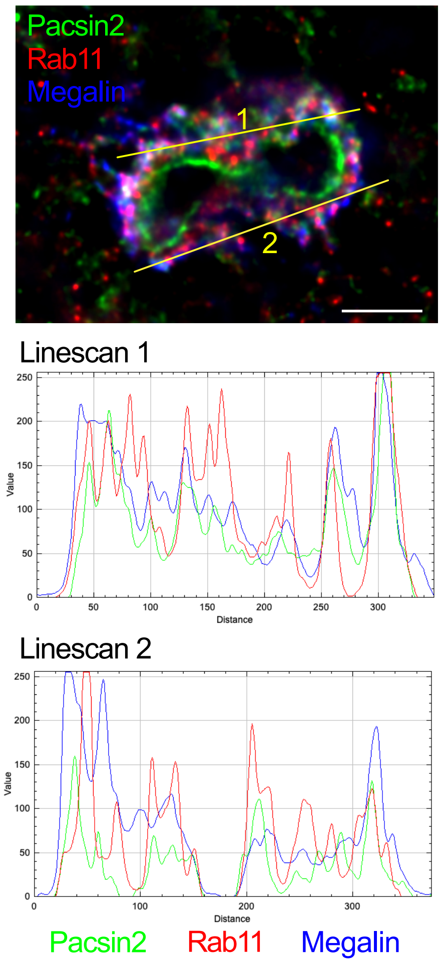

Morgan et al, Fig S5

Fig. S5. Intensity line-scan analysis of pacsin2 immunolabelling in the proximal tubule. Top, transverse cryosection of proximal tubule immunolabeled for Pacsin2 (green), Rab11 (red) and megalin (blue) in 3 dpf wild-type larvae (image taken from Fig 4A and converted to RGB), with positions of the intensity line-scans across the sub-apical region indicated in yellow. Bottom, the plots of signal intensity (y-axis) against relative distance (x-axis) are shown for each line, as indicated. Scale bar, 5 µm.

Biology Open • Supplementary information
